# Living on the edge: precariousness and why it matters for health

**DOI:** 10.1186/s13690-017-0183-y

**Published:** 2017-03-03

**Authors:** Martin McKee, Aaron Reeves, Amy Clair, David Stuckler

**Affiliations:** 1 ECOHOST, London School of Hygiene & Tropical Medicine, 15-17 Tavistock Place, London, WC1H 9SH UK; 2 London School of Economics and Political Science, London, UK; 3 Department of Sociology, University of Oxford, Oxford, UK

**Keywords:** Precariousness, Social determinants of health, Health policy

## Abstract

The post-war period in Europe, between the late 1940s and the 1970s, was characterised by an expansion of the role of by the state, protecting its citizens from risks of unemployment, poverty, homelessness, and food insecurity. This security began to erode in the 1980s as a result of privatisation and deregulation. The withdrawal of the state further accelerated after the 2008 financial crisis, as countries began pursuing deep austerity. The result has been a rise in what has been termed ‘precariousness’. Here we review the development of the concept of precariousness and related phenomena of vulnerability and resilience, before reviewing evidence of growing precariousness in European countries. It describes a series of studies of the impact on precariousness on health in domains of employment, housing, and food, as well as natural experiments of policies that either alleviate or worsen these impacts. It concludes with a warning, drawn from the history of the 1930s, of the political consequences of increasing precariousness in Europe and North America.

## Social and economic security: the post-war consensus

One of the greatest achievements of post-war Western Europe, between the late 1940s and 1970s, was to provide security for its people. One element was collective security from external threats, in the form of the North Atlantic Treaty Organisation (NATO) [1]. The other was security from internal threats, summarised in the United Kingdom by William Beveridge as “the five giant evils” of society: “Want, Disease, Ignorance, Squalor, and Idleness”. The struggle against these giants was the foundation of the post-war British welfare state [2].

The desire for security among politicians and the population in the 1950s and 1960s was understandable. This was a generation that had lived through the depression of the 1930s and the Second World War. Many Europeans had lived with deep uncertainty for many years.

One example was Willem Drees, who served four terms as Dutch Prime Minister in the 1940s and 1950s. He had been imprisoned in Buchenwald at the onset of World War II [3]. Released after a year, he worked with clandestine organisations providing support for the poor, and especially those in the underground and those who lacked links to some of the religious charities that retained support from the collaborationist administration. He was all too aware of the meaning of insecurity, including insecurity of life itself. Immediately after the liberation in 1945 he became minister of social affairs, responsible for creating a new system of old age pensions, using emergency provisions to do so, and greatly expanding the role of the state in providing social assistance.

In the wake of World War II, politicians across Europe from the right and the left, were establishing new programmes and systems of governance that would provide security for their populations, aided by the resources provided by the Marshall Plan and the accompanying forgiveness of debt [4]. In the United Kingdom (UK), the post-war Labour government seized the opportunities provided by the end of the war to create the National Health Service. Nye Bevan, the Minister of Health, launched a compulsory national insurance system that would protect everyone “from the cradle to the grave” [5]. From the other end of the political spectrum, French President Charles de Gaulle resisted pressure from leading industrialists who were opposed to expanding health insurance, pointing out that while the French people had been suffering and, in many cases, dying under the Nazi occupation, the industrialists had been collaborating with the occupiers [6].

Each of these politicians was putting into practice, albeit unknowingly, the situation that the American moral philosopher John Rawls would later describe in his *Theory of Justice* [7]. This involves a group of individuals deciding what a just society would look like. However, unlike the usual situation, in which they create rules and procedures knowing whether they will benefit themselves, these individuals do so from behind a “veil of ignorance”. They make their decisions ignorant of whether they are black or white, rich or poor, male or female, healthy or disabled, or any and more combinations. The resulting society will be one in which access to certain basic freedoms are guaranteed for all, but also that the structures of society must protect the most disadvantaged, ensuring equality of opportunity to take advantage of these freedoms, whatever befalls them and wherever they end up.

By the late 1950s, Europe’s leaders were those who had learned the lessons of the 1930s and 1940s and were determined not to repeat the mistakes of the past. In these ways, the resulting post-war settlement saw the state and employers take on many risks once borne by individuals and families. This newly created social security allowed people to look to the future with confidence, contributing to marked improvements in health and well-being. And this was based on a political consensus. Harold Macmillan, the British Conservative prime minister in the 1950s, boasted that his proudest achievement was building large amounts of social housing [8]. This, in large part, reflected the sense of solidarity that he had developed as an officer in the trenches with his working class soldiers in the first world war [9].

## The retreat of the state

Memories fade. The 1980s were a time of change, at least in the English-speaking world. Ronald Reagan was elected as American President. Margaret Thatcher became British prime minister. Together, they gave their names to a new philosophy, neoliberalism, characterised by a shrinkage in the size of the state, with both privatisation of state owned enterprises, weakening of unions and collective bargaining, and retrenchment of the welfare state [10]. Yet there was a limit. Even Thatcher retained many of the elements of the welfare state and kept many key elements of the economy in public ownership [11]. It was in low and middle income countries where this ideology was pursued with most vigour, in pursuit of the so-called Washington Consensus [12].

It not until was much later that the situation changed in the advanced industrialised countries in Europe. The year was 2008, and the cause was a sequence of events far away [13]. Banks in the United States had accumulated vast cash reserves, including large sums from Chinese investors, and they needed to do something with them. The ingenuity of the bankers knew no bounds. High salaries attracted a new generation, with a new set of skills, into the financial services sector. Tragically, many of the new generation had little idea about what they were doing either.

The large sums of cash that the banks were sitting on were being moved around the world, looking for a home. They found one, in mortgage-backed securities in the United States of America (USA). People who could never have imagined owning their own home now could, by borrowing at unimaginably low rates. The problems began when interest rates rose. Governments gave a very large amount of our money to save the banks, what is now termed “welfare for Wall Street”, leaving none for the ordinary people. Many of these ordinary people had, until then, been coping, but only just.

The situation got worse when their employers started laying people off. Unemployment rose rapidly, placing a burden on social protection systems just at the time when government resources were scarce. The obvious answer was to reduce welfare budgets. In fact, while all countries did cut spending, not all cut welfare [14]. They had a choice and they differed in what they cut. Some did raise taxes, especially those on income and capital gains that would impact most on the rich, including the bankers who had been the beneficiaries of their largesse. However, in many countries, those who had previously enjoyed some degree of job security lost it.

Most of those who lost jobs did find new ones, but the new ones were much less secure [15]. The term ‘zero-hours’ contract entered the vocabulary in some countries. This is exemplified by the contract that British employees of the sandwich chain Subway must sign, stating that “The company has no duty to provide you with work. Your hours of work are not predetermined and will be notified to you on a weekly basis as soon as is reasonably practicable in advance by your store manager. The company has the right to require you to work varied or extended hours from time to time.” These contracts are beneficial to some people, especially mothers returning to the labour market as their children grow up, because they are flexible. However, while these groups do so by choice and have some degree of control over their working arrangements, the rapid rise in zero-hours contracts was seen in those who were underemployed, desiring to work more hours than were available.

What is now emerging is a new class of workers, whose lives are characterised by insecure and unstable employment – or what is referred to as precariousness, which we expand on below.

## The concept of precariousness

French writers studying the nature of work had long recognised that there was a group within the population whose lives could be characterised as precarious, invoking the concept of précarité de l’emploi. In France, this was seen as something to be countered by politicians of both the right, such as Jacques Chirac, and left, such as Francois Hollande. Pitrou, who used the term extensively, identified a set of characteristics that defined précarité [16]. These included some elements related to employment, such as low skills, low wages, and harsh working conditions. However, they also included economic insecurity, inadequate housing, health problems, and lack of social networks. Crucially, precariousness was not the same as poverty, although clearly most people whose lives were precarious were also poor. Other French writers, such as Paugam, have developed this concept further, introducing ideas such as précarité de travail, where the employee is engaged in activities that generate little value, are tedious or repetitive, and attract few rewards, intellectually or financially [17]. More recently, the idea has been extended beyond the conditions of the individual, to describe what is seen as a shift in society to one in which new forms of irregular and uncertain employment dominate [18].

English-speaking writers were also concerned about the changing nature of work. In general, they focussed on what was termed flexibility. While some politicians argued that this was an inevitable accompaniment of technological change and global competition, [19] some, such as Sennett [20] and Reich, [21] were concerned and they too invoked the term precarious.

The situation changed, at least in England, in 2013, with the publication of the results of the Great British Class Survey [22]. This survey included 160,000 predominantly white and predominantly English residents of the UK. It sought to update the Registrar General’s classification of class, introduced in 1923, and which had provided the basis for a wealth of research on social inequalities. The British survey derived class from an individual’s economic, cultural, and social capital. Economic capital was defined as income and assets. Cultural capital was the amount and type of cultural interests and activities. Social capital was the quantity and social status of an individual’s friends, family, and business contacts. It drew heavily on Pierre Bourdieu’s theory of social distinction [23]. It identified seven contemporary classes, starting with the Elite, followed by the Established middle class, the Technical middle class, and then the New affluent workers, the Traditional working class, and finally, the Emergent service sector and, right at the bottom, the Precariat, a term that combined the words precariousness and proletariat. This term was popularised by Guy Standing, who saw it as an inevitable consequence of developments such as information technology and the concentration of power by a small group who accumulated an increasing share of global wealth [24].

Precariousness is a complex, multifaceted concept. Nettleton and Burrows wrote of “the spread of greater labour market flexibility, greater job insecurity, a greater fragility in relationships and a weakening in the formal provision of social welfare” [25]. Beer and colleagues noted how “the concepts of precarious housing and precarious employment make direct reference to the marginal position of many households” [26]. Kalleberg argued that “[precarious] employment…is uncertain, unpredictable, and risky from the point of view of the worker”, [18] while Gill and Pratt argue that “Precariousness (in relation to work) refers to all forms of insecure, contingent, flexible work -- from illegalised, casualised and temporary employment, to homeworking, piecework and freelancing” [27].

What these statements have in common is the view that those whose lives are precarious face uncertainty and risk in several areas, including employment, income, and housing. It is linked to what has been termed the privatisation of risk [28]. The term “precariousness” also features in the literature on these individual issues. For example, the International Labour Organization notes how “precarious work is a means for employers to shift risks and responsibilities on to workers” [29]. For example, researchers on housing policy have noted how mortgage providers seek to transfer risk from themselves to those who are borrowing from them, demanding ever higher deposits for housing purchases, forcing them to spend more, and diverting them to what is in many countries intrinsically insecure private rented sector. Politicians often frame these moves as giving individuals back control, an argument that has intuitive appeal, but which can equally be interpreted as telling people that they are on their own [28].

## Precariousness, resilience, and vulnerability

To understand how precariousness relates to other terms with which it is often used, we turn to the United Nations Development Program (UNDP) 2014 Human Development Report [30]. It notes a “widespread sense of precariousness in the world today-in livelihoods, in personal security, in the environment, and in global politics”. Although precariousness is not actually defined, a search of the report yields many examples of how the term “precarious/precariousness” is used to describe the circumstances in which many people live, including informal employment, threat of conflict, natural disasters, lack of civil, economic and social rights, and exposures to food price hikes. Although the report does not refer to the literature cited above, it is clear that the ideas are closely aligned.

Those whose lives are precarious may be rendered “vulnerable”. This is especially so if they have limited capabilities, for example because they have low reserves of human, economic and social capital. For example, savings or home ownership will, all else being equal, be expected to be protective, as will a supportive social or family network and the means to access justice or other ways to assert their rights. However, even those who do have such capabilities may find them eroded when faced with repeated shocks, with the effect extending beyond the individual to subsequent generations. These capabilities also vary over the life course. People with precarious lives may also become vulnerable because of what is termed restricted social competences. This refers to what social institutions can do.

The UNDP 2014 Human Development Report asks three questions to help understand vulnerability. Who is vulnerable? To what? And Why? For example, the poor, informal workers, and those who are socially excluded are vulnerable to economic and health shocks. Similarly, whole communities may be vulnerable, to conflict and civil unrest, because of low social cohesion, unresponsive institutions and poor governance.

The final term to be considered is resilience. This term is used in different ways in different disciplines but here it is taken to mean “social resilience”, defined as “the capacity of individuals or groups to secure favourable outcomes under new circumstances and, if need be, by new means” [31] while the same idea is captured by Luthar and colleagues, seeing it as the dynamic ability of individuals, communities and entire societies to adapt positively to shocks [32].

In normal circumstances, there is no way of knowing how resilient an individual or a society is. It is only when they are tested by being exposed to a shock that one can possibly know. History provides many examples of people and communities who summoned seemingly superhuman reserves of courage, commitment, and endurance that no one could have anticipated. In contrast, there are many people who aspired to leadership but who failed to rise to the challenge, when it came. It is obvious, given that most people are never tested in this way, that this is an extremely difficult topic to research. Indeed, in the health field, this work has drawn on that of Antonovsky, who tried to understand what was special about those people who survived the concentration camps when so many did not [33]. A systematic review of individual level factors associated with resilience in the face of economic shocks impacting on healthy found evidence to implicate ten separate factors, from gender and marital status to income, attitudes, and social relations [34].

It is important to note that precariousness can cut across traditional classifications of social position or class, based on socio-economic status, employment status, or education. Individuals can be in a state of precariousness even if they are well-educated and in employment-which in conventional social epidemiology would appear favourable-if, for example, that employment is insecure and they have no assets on which to fall back. A contemporary example is provided by junior doctors in the British National Health Service [35]. Although they would seem to be exceptionally privileged, in terms of income and, to some degree, job security, they have no idea, from one week to the next, what hours they will work, or from one year to the next to what part of the country they will be sent. This makes it virtually impossible for anyone with family responsibilities to juggle their multiple commitments. Unsurprisingly, morale is rock bottom, rates of burnout are increasing rapidly, and large numbers are abandoning the profession. This is also a major problem in New Zealand [36].

It is also important to note that precariousness may be perceived, even if not objectively demonstrable. An individual’s perception may be different from the reality, but is nonetheless important and likely to affect their health and well-being. Indeed, while it is clear that job loss is bad for health, there is also considerable evidence that the harmful effects can appear much earlier, coinciding with the anticipation of future problems, regardless of whether those anticipated difficulties ever materialise [37].

In summary, those whose lives are precarious are at greater risk of a shock, such as job or housing loss, and if it occurs they are also at risk of a cascade of events over which they have little control, and few reserves on which to draw, thereby reducing their resilience.

## The state as a protector

The institutions of society and, in particular, government, can reduce the numbers of people whose lives are precarious and protect them from its consequences, reducing the risk of a shock and by mitigating its effects should it occur, for example by creating safety nets. As Vives and colleagues note, “a strong welfare state protects workers” from the consequences of employment precariousness [38]. This lesson has been relearned during the global financial crisis that began in 2008. The crisis has had profound consequences for health (Table 1) [39] but the responses that have been adopted have provided many natural experiments, both good and bad, that shed light on the health consequences when those whose lives are precarious face a shock, such as the loss of a job or a home. In the following sections, we explore how the institutions of the state either protect or, in some cases, fail to protect those who face precariousness in relation to employment, work, housing, and food security.

**Table 1:** The health effects of the financial crisis

The health effects of the global financial crisis have been complex. Some causes of death have declined, in particular road traffic injuries, as the volume of heavy traffic on the roads diminished. But others increased, such as suicides [72–74]. Yet this was not inevitable. The historical record reveals that there was a clear link between job losses and suicides in some countries, such as Spain. Yet in others, such as Sweden, there was no such link. The difference was the strength of the welfare state, and specifically investment in what are called active labour market programmes [75, 76]. These are programs that help people get back into work, providing information and retraining and support for people with disabilities. But more fundamentally, they are a means by which the state can say that it cares about its people.
The situation with infectious disease was especially complex [77]. First, the circumstances had to exist to rely a particular infection to emerge or re-emerge. It was not plausible that malaria would emerge in Norway. Second, there had to be a breakdown in those processes that had kept it under control. Thus, cutbacks in vector control allowed malaria to reappear in Greece. Cuts to a needle exchange program in Athens contributed to an epidemic of HIV [78]. Abandoned swimming pools following mortgage foreclosures in California provided breeding grounds for the mosquitoes transmitting West Nile virus.

## Précarité de l’emploi –precariousness of employment

A study of the impact of job loss on mental health among Greeks in 2009, before the worst of the austerity package, and in 2011 when it was in full force and when many more Greeks were living lives that were precarious, found that job loss was associated with deteriorating mental health in both periods but the impact was very much greater in the context of austerity [40]. Thus, both vulnerability, or the probability of losing one’s job, and the adverse effects that resulted because of a loss of social resilience, were exacerbated by the wider economic situation.

Yet there is evidence that policies which reduce precariousness are beneficial against such hazards. Theresa May, the British Prime Minister, has made much of her desire to help those she describes as just about managing. One reason that many people are just about managing is that they are paid very little. This means that, if misfortune should befall them, they will have few if any financial resources to fall back on. Yet, some years ago, the situation was even worse as there was no minimum wage. But can an increase in a few pence make a difference? The introduction of the minimum wage in the UK, in 1999, was a natural experiment [41]. A study using longitudinal data tracked three groups of people. The first was those who were below the threshold and who saw an increase in their income. The second comprised those who were just above it, and so derived no benefit. The third comprised those who were below it and stayed there because the policy was inadequately enforced. There was a significant improvement in mental health, but only among those whose incomes increased, and thus whose lives were marginally less precarious. Although they only had a few more pence each hour, the size of the effect was considerable, equating to that seen among people prescribed anti-depressants. For some people, a small change can make a big difference.

## Précarité de travail – precariousness of work

France has experienced an epidemic of workplace suicides, with increasing numbers of employees choosing to kill themselves in the face of extreme pressures at work [42]. Suicides have affected a wide range of companies and sectors including postal services, car manufacturing, telecommunications, electricity and gas, banks, supermarkets, research centre and call centres. In a number of cases, individuals have left letters, subsequently published in the press, in which they explicitly blame work or conditions at work as the cause of their actions. Others have chosen to kill themselves in a highly visible or symbolic way, returning to work to take their own lives, for example by hanging themselves in their offices, to make clear the connections between their suicide and work. In July 2016, Paris prosecutors announced that the former chief executive of the telecoms provider, France Télécom, now rebranded as Orange, and six senior managers may face criminal charges in relation to suicides among its employees. This follows an earlier case when a French court of appeal found the car manufacturer, Renault, guilty of gross negligence regarding three suicides at the company.

At least the French government has recognised that this is a problem. Any suicide at work is considered work-related until proven otherwise, and suicides outside work are investigated as work-related if family members can show evidence suggesting a link. In contrast, in the United Kingdom, even those suicides committed in the workplace are presumed to be individual and voluntary acts and the relevant legislation states that ‘All deaths to workers and non-workers, with the exception of suicides, must be reported if they arise from a work-related accident’ [43].

## Precarité de lodgement – precariousness of housing

We now look beyond Paugam’s writing on employment and work, although for consistency we retain the use of French terminology. People may also feel precarious because of concerns about having somewhere to live. This was apparent in a study in Spain early in the financial crisis. This used data on patients attending primary care centres in 2006–07 and 2010–11, before and during the economic crisis [44]. All completed a standardised instrument designed to diagnose mental disorders. There was a significant increase in mental illness, after adjusting for the usual socio-demographic confounders. In particular, there were large increases in depression and anxiety and alcohol-related disorders. As might be expected, job loss was a major factor but so was getting into housing arrears or the threat of eviction, independent of employment status.

That study had several limitations. It was based on two cross sectional surveys, so we were not following up individuals. There was also limited information about the individual circumstances of respondents. At the time, there were very few data following up individuals during the period of the crisis. By about 2014 this changed and the data from the EU’s Survey of Income and Living Conditions became available for the early years of the crisis [45]. Although it contained very few questions on health, there were some, opening many new opportunities do.

Another study of housing identified all respondents from the then 27 EU member states who had no housing arrears in 2008 and followed them to 2010, when many more were facing situations that were precarious because of job losses and cuts to social protection [46]. Those transitioning into arrears experienced a deterioration in their mental health, but only if they were renting their accommodation. Those who owned their accommodation experienced no deterioration, after adjusting for other factors, consistent with the literature suggesting that capabilities, such as ownership of assets, reduces vulnerability. Crucially, the effect of falling into rent arears was independent of, and greater than job loss. Once again, the effect varied among countries. In some, people were relatively protected. In others, such as Belgium, Austria and Italy, the effect was substantial.

A natural experiment in the UK offered an opportunity to look a little closer at housing and health, in April 2011, when the government reduced financial support for low income persons renting in the private sector [47]. The effect was substantial, with those receiving housing benefit losing, on average, about €1,500 a year, greatly increasing their precariousness. The prevalence of health problems was compared in those receiving housing benefit, who would suffer a loss, and those who not receiving it, who would be unaffected. Given that this was amid the economic crisis, it was unsurprising that even those spared these specific cuts experienced some worsening in mental health. However, the change was several times greater among those whose housing benefit was cut.

It is, of course, important to look upstream, to ascertain the causes of the causes. Why do some people experience housing problems and others not? There are clearly many individual factors, but are there aspects of government policy that play a role, placing more people in situations that are precarious in some areas rather than others? One study looked at this within a single country, England, seeking to explain variations in homelessness claims between 2004 and 2012 [48]. As expected, reductions in the economic activity in a local area were important. These led to job losses and reductions in income, with lower spending impacting on local shops and service providers. But homelessness was also associated with reductions in welfare spending, and especially cuts to housing services and payments, as expected, but also social care and income support for older people.

## Precarité de la sécurité alimentaire – precariousness of food security

The final area related to precariousness is the ability to feed oneself and one’s family. A study using data from 21 countries revealed that food insecurity had increased between 2004 and 2012 and this was associated with both job loss and income reduction [49]. But, again, where robust social protection policies were in place, the impact of rising unemployment or stagnating wages on food insecurity was reduced. In countries without adequate safety nets, people have relied on charitable organizations, such as foodbanks, to obtain food. In the UK, for example, there has been a very large increase in the number of foodbanks and those using them [48]. Ministers have attributed it to people being unable to manage their finances, or spending their money of alcohol and cigarettes. Because the food is free, it is assumed there is infinite demand. Yet these politicians seem not to realise that people can only use a foodbank if they are referred, typically by a doctor or social worker. One study showed that the growth of foodbanks followed job losses, cuts in welfare spending, and what are termed sanctions, which temporarily stop welfare payments to those claiming them [48]. Sanctions are deeply troubling because they appear to disproportionately affect disabled people and lone parents, [50] pushing them to rely on the charity of others or, in some cases, petty crime to feed their families [51, 52]. The government claims that sanction encourage people back into work but there is no evidence to support this [53].

To summarise so far, the financial crisis and the subsequent imposition of austerity have impacted on people in many ways. An estimated 5 million EU citizens lost their jobs between 2008 and 2010. Many others experienced reductions in income. Some lost their homes and while there is no system for collecting comparable data, surveys in countries such as Spain and the UK suggest that numbers of homeless increased by about 15% between 2008 and 2010. Others went without food. Their lives became more precarious. This meant that not only were they at greater risk of misfortune but the consequences were worse when they experienced it.

This is just the tip of the iceberg. Many who have escaped these experiences live in constant fear of the future. Their jobs and income may be secure for now, but for how much longer? They can still afford their homes, but will this continue? And if they must move, what will this mean for getting to work, for their social support networks, and for their children’s schooling?

## Beyond health: the political consequences of precariousness

These findings show why health professionals should be concerned about precariousness, as it impacts on the health of some of the most vulnerable people in societies. But there is another reason why society should be concerned about the growth and persistence of a section of the population who feel left behind, in a world characterised by uncertainty.

The word precarious is related to the Latin, precor, to beseech or to pray. Once, in the days when, to quote Thomas Hobbes, life was nasty, brutish and short, those whose lives were most precarious were likely to turn to religion [54]. Some still do. But, at least in the twentieth century, there were times when they turned to others who promised a better future.

A recent study asked whether the austerity implemented during the Weimar Republic contributed to the rise of National Socialism [55]. This was a time of great uncertainty. Unemployment rose steeply. Rampant inflation destroyed savings. Many Germans emigrated, in search of a better life in the new world. Using data on voting patterns in the five Reichstag elections between 1928 and 1933 and on a variety of measures of the economy, including government spending and tax withheld from wages, hourly wages and economic output, it was possible, by means of a geographical analysis of patterns in small administrative areas, to construct a dataset at the level of constituencies. The findings revealed a clear association between the depth of austerity and the rise in support for the National Socialists. Crucially, this was not simply the result of impoverishment. The very poor, a group that was hit hard by job losses, tended to turn to the communists. It was those just above them in the pecking order who turned to the Nazis, the group who had something to lose.

Of course, great care is needed in drawing parallels with the events of the 1930s and 1940s in Europe. It is almost unthinkable that the events of those years could ever be repeated. But 2016 has surely shattered any remaining complacency. In June, the United Kingdom saw a small majority vote to leave the European Union, encouraged by politicians who argued that the British public had had enough of those “experts” who warned, correctly, of the profoundly damaging consequences of doing so. Donald Trump has been elected as President of the United States, despite clear evidence of his unsuitability, intellectually and temperamentally, for this position of enormous responsibility.

These concerns are not confined to the Anglo-Saxon world. Across Europe, parties attacking what is portrayed as an out of touch establishment are attracting growing support [56]. It now seems likely that Marine Le Pen will reach the 2nd stage of the 2017 French presidential election. In Germany, Alternative für Deutschland is adopting a blatantly racist strategy. Elsewhere there is the Swiss People’s Party, the Swedish Democrats, the True Finns, Jobbik in Hungary, Golden Dawn in Greece and Geert Wilders and his Party for Freedom in The Netherlands. The historical parallels are impossible to ignore, with many of these parties using language reminiscent of the 1930s. Incredibly, a few have even adopted symbols that draw explicitly on those of the Nazi era, including variants of the swastika. In the USA, Donald Trump has attracted support from not only from the Ku Klux Klan but also from groups that identify explicitly with the Nazis. One British activist on social media, who closely observes the Daily Mail, a widely read tabloid newspaper notorious for its support of the Nazis in the 1930s, posted comments on its articles online, quoting verbatim from Mein Kampf and Der Stürmer, substituting words related to migrants and Muslims for Jews [57]. His comments received many “likes” and positive comments from the newspaper’s readership. And many in the UK were shocked by a UKIP (United Kingdom Independence Party) poster picturing a line of migrants using the same imagery as the Nazis. Donald Trump, when asked repeatedly what was the difference between his proposals to register Muslims in the United States and the Nazi registers of Jews could only reply “you tell me” [58].

Inevitably, scholars have sought to explain what is happening, in some cases drawing on the Marxist concept of false consciousness [59]. Recent analyses have focused on the United States, including both scientific studies and popular books. Among the most readable is Thomas Franks’ book “What’s the matter with Kansas?”, [60] exploring how conservatives have focused attention on issues such as abortion and immigration as a means of distracting attention from policies that damage those whose votes they need to be elected, the white working class, while Westen has examined this issue from the perspective of psychology, exploring the role of cognitive biases [61]. Others have looked at the role of the media, and in particular the impact of Fox News. As Al Franken, now the junior senator from Minnesota, has noted, its coverage is anything but fair and balanced [62]. Moreover, as one elegant study relating voting patterns to its rollout on cable showed, it does have an impact on voting behaviour. The messages promoted by much of the media in the UK during the debate on Brexit were not only blatantly dishonest, although in this they were simply repeating unquestioningly what some of the more disreputable politicians were saying, but were also clearly racist. Subsequently, it has been shown that Rupert Murdoch’s Sun newspaper can also shift voting behaviour, even when it does not change underlying values [63].

Lipset [64] and Bell [65], in their classic studies of the rise of fascism in Weimar Germany, Poujadism in France, and McCarthyism in the United States, noted that support for these extremist parties was concentrated among the petty bourgeoisie, the small business people, shopkeepers, and independent farmers who had achieved comfortable lives but saw it threatened by others, such as Jews, organised labour, and communists. Lipset noted that “extremist movements have much in common. They appealed to the disgruntled and psychologically homeless, to the personal failures, the socially isolated, economic league insecure, the uneducated, unsophisticated, and the authoritarian persons” [66].

These findings are borne out by research in the wake of the U.K.’s referendum. Those most likely to vote for leave were the poor and unemployed, those looking for work, people with low skills and those in precarious manual occupations [67]. They are also supported by recent research in Germany, which describes associations between increased precariousness of work, greater social insecurity and right-wing populist orientations [68].

There is an emerging group within many populations that feel disconnected from what they see as a distant establishment. Many of the certainties that they took for granted, such as jobs for life, ever improving living conditions, and children whose prospects were better than their own seem to have vanished. They look around for someone to blame and they do not need to look far. In the shops, in the streets, and in the schools they see people who look different. And when politicians also point the finger of blame at those who look different, it is far too easy for an increasingly precarious populous to accept this narrative. What they do not see, of course, is that these others are doing the jobs that they neither want nor have the skills to do. They forget that their health care systems manage only because they import skilled workers from the rest of the world. They forget that their elderly relatives are looked after by migrants. And the newspapers they read conveniently overlook the evidence that migrants make a positive contribution to their economies. For anyone with a sense of history, it should be deeply worrying. The vote in the UK should surely be a warning that nothing can be taken for granted. Many thought that it was inconceivable that so many of the British people would vote against their own interests in the EU referendum, but they did. The author Charles Emerson has written a superb account of life in the year 1913 in almost 30 cities across the world [69]. What is striking is that for almost all of those whose stories are told, the carnage of the following 4 years was equally inconceivable.

Anyone who believes in the enlightenment values of evidence and enquiry, of tolerance and mutual respect, and the more recent value of solidarity cannot ignore those factors that are driving politics today. And this means understanding the lives of those who see the world in a very different way from academic researchers and policy makers.

## Conclusion

Those concerned about the health of the population, and especially those most disadvantaged, must try to understand the impact on health of the changes that are taking place in society. Europe offers an incredible natural laboratory to study these issues. The growth in precariousness is not inevitable. While wages have stagnated in the UK and Germany, they have risen in Finland and Slovakia. Meanwhile, skyrocketing housing prices in the UK housing contrasts with The Netherlands, which maintained stable housing prices, even during the recessions of 2008. Sweden has slowly reduced pension support, but France has increased it for some older people. There are many different responses that can be learnt from. Some, as the Danish model of ‘flexicurity’, provide market freedom in employment but compensated by generous secure benefits, [70] creating a situation where one can experience job insecurity, but relatively low labour market insecurity, with confidence in finding another job and sufficient severance pay. Others, such as the German system of rent and tenure controls, provide security in the housing market [71]. All have strengths and weaknesses but they offer many opportunities to learn from.

Yet there is another reason to be concerned. Democratic systems are based on a social contract. And those with power should not use it to breach that contract. That can, as can be seen from history, have consequences for everyone.

## Abbreviations

NATO: North Atlantic treaty organisation
UK: United Kingdom
UKIP: United Kingdom independence party
UNDP: United Nations development programme
USA: United States of America
